# Evaluation of the relationship between Demodex infestation and obesity in rosacea patients

**DOI:** 10.1111/jocd.16417

**Published:** 2024-05-30

**Authors:** Esra Kiratli Nalbant, Ilknur Balta, H. Meral Eksioglu

**Affiliations:** ^1^ Ankara Training and Research Center, Department of Dermatology and Venerology Health Sciences University Ankara Turkey

**Keywords:** Demodex, Demodex infestation, demodicosis, obesity, rosacea

## Abstract

**Background:**

The changes in the skin caused by obesity may predispose patients with rosacea to *Demodex* infestation (DI). Besides, identifying predisposing factors in these patients is important for initial treatment plan.

**Aims:**

The main purpose of this study is to determine the relationship between DI and obesity in patients with rosacea.

**Methods:**

Sociodemographic characteristics, body mass index, and clinical features of 130 rosacea patients were recorded. *Demodex* density was measured by noninvasive standard skin biopsy, and a determination of ≥5/cm^2^ mites was regarded as positive for DI. Patients with a BMI of 30 and above were categorized as the obese group, and those with a BMI below 30 were classified as the nonobese group. Moreover, patients were divided according to DI into positive and negative. These groups were compared with each other.

**Results:**

Upon comparing the patients with and without obesity, it was noted that the obese patients had significantly higher rates of erythematotelangiectatic type rosacea, rosacea severity, and flushing complaints. Furthermore, results show that the DI was significantly more common in obese patients. The most determinant factors for predicting DI in rosacea patients were papulopustular type rosacea, obesity, and rosacea severity, respectively.

**Conclusions:**

In this study, DI was found to be more common in obese patients with rosacea. Besides, obesity was found to be one of the most determining factors in detecting DI in patients with rosacea.

## 1 INTRODUCTION

Rosacea, characterized by papules, pustules, erythema, telangiectasia, and edema mainly in the midface area, is a very common chronic inflammatory skin disease affecting a significant proportion of people.^1^ While the pathogenesis of rosacea is still not fully understood, it is suggested that neurogenic inflammation and neurovascular changes, innate and adaptive immune response changes, and colonization of microorganisms such as *Demodex folliculorum* and *Demodex brevis* in the skin are involved in the pathogenesis.^2^

Adult *Demodex* parasites are 0.2–0.4 mm arthropods living in the pilosebaceous follicles of normal skin. These parasites can cause tissue damage mechanically with their sharp mouths and tarsal claws and chemically with the proteases they produce. Additionally, their exoskeletons consist primarily of a known inflammatory stimulus, chitin.^3^ Although at low densities, mites are accepted as members of the normal human skin microbiota, deterioration of some physical barrier properties of the skin due to various reasons such as skin diseases, improper skin cleaning, and topical drug use, may cause these mites to begin to multiply in the skin and stimulate inflammation in various ways.^4^ The diagnosis of *Demodex* infestation (DI) in humans is made by detecting an increase in mite count (≥5/cm^2^) in active lesions. It is considered that in patients with rosacea, increased numbers of *Demodex* parasites block the pilosebaceous follicle, causing a rupture in the follicular epithelium and follicular damage.^5^ Despite suggestions that the inflammation pathway in rosacea starts with this damage, the role of parasites still needs further explanation.

Nowadays, obesity is an increasing, global public health issue. Along with its well‐known harmful impacts on internal organs and psychology, many studies examine its effects on the skin. Studies are reporting that obesity disrupts the barrier function of the skin and causes many pathologies in sebaceous and sweat glands, subcutaneous tissue, and wound healing.^6^, ^7^, ^8^ Apart from the physiological alterations that obesity brings to the skin, research has been published in the literature that indicates obesity exacerbates the opportunistic skin parasitosis known as DI by modulating the immune system.^9^

Identifying which additional factors predispose rosacea patients to DI is very important for the treatment plan of these patients. One of these factors may be obesity due to the effects mentioned above. This study primarily aims to examine the relationship between DI and obesity in patients with rosacea and to determine whether obesity is a risk factor for DI. The secondary aim of this study is to detect markers of demodicosis in patients with rosacea.

## 2 MATERIALS AND METHODS

The study was reviewed and approved by the local ethics committee (protocol no:48865165‐020, date of approval:01/22/2018), and all participants gave written informed consent. The study was carried out according to the principles expressed in the Declaration of Helsinki.

### 2.1 Participants

The study was designed as a descriptive, cross‐sectional prospective study from a single center, Ankara Training and Research Hospital, Dermatology clinic. All consecutive patients aged 18 and over; who applied to our clinic between February 2020 and July 2020 and diagnosed with rosacea for the first time were examined. Exclusion criteria for this study are having a communication disability, being pregnant, or in a breastfeeding period, having a psychiatric disease or systemic diseases that may affect the immune system (malignancy, immunodeficiency, autoimmune, cardiac, metabolic, or endocrine diseases), having facial dermatosis other than rosacea and using systemic or topical corticosteroids, immunosuppressive drugs or topical antibiotics on the face for any reason within the previous month. The study was terminated when a sufficient number of patients was reached. In total, 130 rosacea patients were included in the study.

All patients' height and weight measurements were taken and their body mass index (BMI) values were calculated using the formula kg/m^2^. BMI values were categorized as: <18.5 kg/m^2^ underweight, 18.5–24.9 kg/m^2^ normal, 25–29.9 kg/m^2^ overweight, 30–34.9 kg/m^2^ class I obese, 35–39.9 kg/m^2^ class II obese, and >40 kg/m^2^ as class III, morbidly obese. Demographic data, disease duration, triggering factors, rosacea clinical subtype, and disease severity of all patients were also recorded. The severity of rosacea was determined according to the severity scale defined by the National Rosacea Society Expert Committee.

### 2.2 Procedure

*Demodex* density was determined using the standard superficial skin biopsy (SSSB) method.^10^ The researchers applied a slide dripped with cyanoacrylate glue to the lesioned area of the patients. They gently pulled the slide out and examined it under a microscope. The *Demodex* density in 1 cm^2^ was measured. The presence of five or more *Demodex* in 1 cm^2^ was regarded as ‘positive’ for DI.

### 2.3 Statistical analysis

Firstly, a total of 130 patients (88 females, 42 males) included in the study were divided into two: 58 obese (BMI ≥30) and 72 nonobese (BMI <30) patients. Statistical evaluation was first made by comparing these two groups. Then, all patients were separated into two groups again, according to DI positivity. Statistical analysis was also performed by comparing these two groups. In order to determine the best predictor(s) which effect on the presence of DI was investigated by multiple logistic regression analysis via the Backward LR procedure‐any variable whose univariable model along with all variables of known clinical importance. Odds ratios, 95% confidence intervals, and Wald statistics for each independent variable were also calculated.

## 3 RESULTS

### 3.1 Demographic and clinical characteristics

Of 130 subjects, 42 males (32.3%) and 88 females (67.7%) were included, and the mean age and BMI of rosacea subjects were 47.7 ± 12.8 and 29.8 ± 5.9, respectively. Of all patients, 72 (55.4%) were nonobese, 30 (23.1%) were normal, 42 (32.3%) were overweight, 58 (44.6%) were obese, 38 (29.2%) were class I obese, 13 (10.0%) were class II obese, and 7 (5.4%) were class III obese. The average disease duration was found to be 3 years (min 1–max 30 years). Considering the clinical subtypes of rosacea, 69 (53.1%) of the patients were erythematotelangiectatic (ET), 54 (41.5%) were papulopustular (PP), 3 (2.3%) were ocular, and 4 (3.1%) were phymatous. As classified according to disease severity, 61 of the patients were in the mild group (46.9%), 56 in the moderate group (43.1%), and 13 in the severe group (10%). Of the patients, 24 (18.5%) had a family history of rosacea, and 56 (43.1%) were found to be positive for DI. (Table 1).

**TABLE 1:** Study population characteristics, *n* = 130.

Age (y) 9,7, mean ± SD	47.7 ± 12.8
Gender, *n*, (%)	
Male	42 (32.3)
Female	88 (67.7)
Height (cm), mean ± SD	1.62 ± 0.076
BMI (kg/m^2^), mean ± SD	29.8 ± 5.9
BMI distribution, *n*, (%)	
Normal	30 (23.1)
Overweight	42 (32.3)
Obese	58 (44.6)
Class I	38 (29.2)
Class II	13 (10.0)
Class III	7 (5.4)
Disease duration (y), range	3 (1–30)
Rosacea subtype, *n*, (%)	
ETR	69 (53.1)
PP	54 (41.5)
Phymatous	4 (3.1)
Ocular	3 (2.3)
Disease severity, *n*, (%)	
Mild	61 (46.9)
Moderate	56 (43.1)
Severe	13 (10.0)
Family history, *n*, (%)	24 (18.5)

Abbreviations: BMI, Body mass index; ETR, erythematotelangiectazic; PP, papulopustular; SD, standard deviation.

When obese and nonobese groups were compared, there were no significant differences in terms of age, gender, alcohol consumption, family history of rosacea, disease duration, make‐up habit, or shared towel use (*p* > 0.05). On the contrary, smoking history was more frequent in the nonobese group than the obese group (*p* = 0.024). In addition, there was a statistically significant difference between the groups regarding the distribution of rosacea types (*p* = 0.020). We observed that the nonobese group had more PP‐type rosacea (PPR), while the obese group had more ET‐type rosacea (ETR) (*p* = 0.045 and *p* = 0.028). When rosacea complaints were evaluated, flushing was more common in the obese group (*p* = 0.028). The severity of rosacea was also significantly higher in the obese group (*p* = 0.014). Besides, DI was statistically significantly more common in the obese group than the nonobese group (*p* = 0.002). (Table 2).

**TABLE 2:** Comparison of obese and nonobese study groups.

	Nonobese group (*n* = 72)	Obese group (*n* = 58)	*p* Value
Age (years), mean ± SD	46.1 ± 12.0	49.7 ± 13.6	0.113^a^
Gender, *n*, (%)			
Male	24 (33.3)	18 (42.9)	0.85^b^
Female	48 (66.7)	40 (57.1)	
Smoking habit, *n*, (%)	20 (27.8)	6 (10.3)	0.024^b^
Alcohol consumption, *n*, (%)	7 (9.7)	2 (3.4)	0.297^d^
Family History, *n*, (%)	15 (20.8)	9 (15.5)	0.583^b^
Disease duration, (y), range	2 (1–30)	12.5 (1–30)	0.322^c^
Rosacea subtype, *n*, (%)			
ETR	32 (44.4)*	37 (63,8)*	0.020^e^
PP	36 (50.0)**	18 (31.0)**	
Phymatous	1 (1.4)	3 (5.2)	
Ocular	3 (4.2)	0 (0.0)	
Rosacea severity, *n*, (%)			
Mild	40 (55.6)	21 (36.2)	0.014^a^
Moderate	29 (40.3)	27 (46.6)	
Severe	3 (4.2)	10 (17.2)	
Rosacea symptoms, *n*, (%)			
Flushing	43 (59.7)	46 (79.3)	0.028^b^
Oedema	25 (34.7)	29 (50.0)	0.115^b^
Itching	39 (54.2)	42 (72.4)	0.051^b^
Persistent erythema	33 (45.8)	36 (62.1)	0.065^a^
Burning‐stinging sensation	52 (72.2)	37 (63.8)	0.402^b^
D (+), *n*, (%)	22 (30.6)	34 (58.6)	0.002^b^

Abbreviations: ETR, erythematotelangiectazic; PP, papulopustular; SD, standart deviation.

*Note*: D (+): The patients diagnosed with demodex infestation.

^a^ Pearson chi‐square test.

^b^ Continuity correction chi‐square test.

^c^ Mann–Whitney U test.

^d^ Fisher's exact test.

^e^ Fisher–Freeman–Halton test.

*p = 0.028 versus **p = 0.045.

When all patients were divided according to DI positivity and compared, statistically significant differences were found in terms of age (*p* = 0.44), obesity (*p* = 0.02), rosacea subtype (*p* = 0.04), severity (*p* < 0.01), pruritus (*p* = 0.40), and persistent erythema (*p* = 0.10) (Table 3). The average age of the DI (+) group was statistically significantly higher than that of the DI (−) group. We observed that the DI (+) group had more PPR, while the DI (−) group had more ETR. (*p* = 0.003 and *p* = 0.010). When both subtypes were compared, DI positivity was more common in PPR than in ETR (*p* = 0.004).

**TABLE 3:** Comparison of the demographic and clinical features of patients according to demodex infestation positivity.

	D (−) (*n* = 74)	D (+) (*n* = 56)	*p* Value
Age (y), mean ± SD	45.8 ± 12.6	50.3 ± 12.8	**0.044** ^a^
Gender, *n*, (%)			
Male	18 (24.3)	13 (23.2)	>0.999^b^
Female	56 (75.7)	43 (76.8)	
Obesity, *n*, (%)			
Non‐obese	50 (67.6)	22 (39.3)	**0.002** ^b^
Obese	24 (32.4)	34 (60.7)	
Disease duration (y), range	4 (1–30)	3 (1–30)	0.627^d^
Family history, *n*, (%)	15 (20.3)	9 (16.1)	0.702^b^
Rosacea subtype, *n*, (%)			
ETR	47 (63.5)*	22 (39.3)*	**0.004** ^c^
Phymatous	2 (2.7)	2 (3.6)	
Ocular	3 (4.1)	0 (0.0)	
PP	22 (29.7)**	32 (57.1)**	
Rosacea severity, *n*, (%)			
Mild	46 (62.2)	15 (26.8)	**<0.001** ^e^
Moderate	25 (33.8)	31 (55.4)	
Severe	3 (4.1)	10 (17.9)	
Rosacea symptoms, *n*, (%)			
Flushing	51 (68.9)	38 (67.9)	>0.999^b^
Oedema	31 (41.9)	23 (41.1)	>0.999^b^
Itching	40 (54.1)	41 (73.2)	**0.040** ^b^
Persistent erythema	32 (43.2)	37 (66.1)	**0.010** ^e^
Burning‐stinging sensation	49 (66.2)	40 (71.4)	0.658^b^

Abbreviations: ETR, erythematotelangiectazic; PP, papulopustular.

*Note*: D (−) The patients without demodex infestation, D (+) The patients with demodex infestion.

^a^ Student's *t* test.

^b^ Continuity correction chi‐square test.

^c^ Fisher–Freeman–Halton test.

^d^ Mann–Whitney U test.

^e^ Pearson's chi‐square test.

*p = 0.010 versus **p = 0.003. *p* < 0.05 values are written in bold.

Besides, the severity of rosacea was significantly higher in the DI (+) group (*p* < 0.001). While the complaints of itching and persistent erythema were more common in the DI (+) group, the frequencies of other complaints were similar between the groups. (Table 3).

### 3.2 Analysis of the best predictors of *Demodex* infestation in rosacea patients

Variables determined to be *p* < 0.10 as a result of univariate statistical analysis were included in the regression model as candidate factors. As a result of the retrospective stepwise elimination method, the most determining factors to predict the presence of DI were PPR, obesity, and rosacea severity. Regardless of other factors, having PPR increased the prospect of detecting DI 4.410 times. (95% Cl: 1.773–10.966 and *p* < 0.001).

Obesity and the increase in the severity of rosacea also increased this probability 4.335 times (95% Cl: 1.746–10.765 and *p* = 0.002) and 2.539 times (95% Cl: 1.312–4.914) (*p* = 0.006), respectively. (Table 4).

**TABLE 4:** Determination of the predictors for the presence of demodex infestation by logistic regression analysis.

	Odds ratio	%95 Confidence Interval		Wald	*p* Value
		Lower limit	Upper limit		
Model 1					
Age	1.016	0.982	1.050	0.811	0.368
Obesity	3.877	1.518	9.903	8.019	**0.005**
Rosacea severity	2.022	0.995	4.112	3.784	0.052
PP	4.798	1.842	12.497	10.307	**<0.001**
Itching	1.711	0.721	4.060	1.487	0.223
PE	1.893	0.775	4.626	1.961	0.161
Model 2					
Obesity	4.095	1.620	10.351	8.881	**0.003**
Rosacea severity	1.983	0.983	4.003	3.652	0.056
PP	5.067	1.961	13.089	11.230	**<0.001**
Itching	1.725	0.730	4.071	1.546	0.214
PE	2.001	0.828	4.838	2.374	0.123
Model 3					
Obesity	4.422	1.764	11.085	10.051	**0.002**
Rosacea severity	2.095	1.046	4.195	4.359	**0.037**
PP	5.054	1.964	13.005	11.288	**<0.001**
PE	1.971	0,819	4.746	2.293	0.130
Model 4					
Obesity	4.335	1.746	10.765	9.993	**0.002**
Rosacea severity	2.539	1.312	4.914	7.651	**0.006**
PP	4.410	1.773	10.966	10.192	**<0.001**

Abbreviations: PE, persistent erythema; PP, papulopustular. p< 0.05 values are written in bold.

## 4 DISCUSSION

The possible role of *Demodex* mites in the pathogenesis of rosacea is yet to be fully elucidated. However, many studies suggest strong relationship between DI and rosacea.^11^, ^12^, ^13^ In order to understand this relationship, the demographic and clinical findings of rosacea patients with DI must be intricately investigated. This study mainly shows that obesity is also one of the factors that might play a role in this relationship.

Under normal circumstances, *Demodex* mites live on the skin without causing any harm to the host. It is still unclear why these mites multiply and cause skin disease in some people. However, some individual and environmental factors are thought to predispose to it. Innate and adaptive immune response disorders could be one of these predisposing factors. Akilov et al. found that immunological responses to *Demodex* vary with HLA types, with some phenotypes showing higher susceptibility. Specifically, the HLA‐Cw2 phenotype exhibited a susceptibility to infestation, with a decrease in the number of CD3+, CD8+ T cells, and NK cells, and a deterioration in leukocyte functions. In contrast, the HLA‐A2 phenotype demonstrated resistance.^14^ Besides, there are reports of more DI in diseases such as diabetes mellitus,^15^ chronic kidney disease,^16^ hematological malignities,^17^ HIV infection,^18^ and malnutrition^19^ due to the suppression of the immune system.

Obesity has numerous negative effects on internal organs and systems, including the immune system. It can affect both cellular and humoral immunity, increasing susceptibility to infections. It disrupts lymphoid tissue architecture and leukocyte development, decreasing the number and diversity of T cells and causing disorders in their activation and differentiation.^20^ Studies have demonstrated an increase in proinflammatory cytokines and the number of CD4+ T cells in the peripheral blood of obese individuals, accompanied by a decrease in CD8+ T cells.^21^ It also impairs wound healing and the barrier function of the skin by disrupting collagen structure and function while increasing sweat and sebaceous gland activity.^7^ These changes in immunity caused by obesity may make individuals susceptible to DI. The decrease in CD8+ T cells, in particular, may be significant regarding DI susceptibility. Another factor that enables the proliferation of parasites may be the increased sebum production in obese individuals' skin, which provides a food source for *Demodex* mites.^22^ In a study conducted by Dokuyucu et al., it was found that the BMIs of patients with DI were higher than those of patients without detection. These authors showed that the increase in the incidence of DI was parallel to the increase in BMI.^9^ We examined 130 rosacea patients and found that DI incidence was significantly higher in obese rosacea patients.

Our study created a comprehensive profile of rosacea patients with DI, identifying older age, PPR, obesity, severe rosacea, and complaints of itching or persistent erythema as key features. Previous literature supported these results^3^, ^23^, ^24^, ^25^ except we found no association between flushing and DI, despite recent studies suggesting *Demodex* mites play a role in the etiopathogenesis of flushing in rosacea.^26^ On the contrary, we found flushing complaints were more common in obese patients. These findings suggest that multiple factors, rather than a single cause, contribute to the etiopathogenesis of flushing. Regression analysis indicated that PPR, obesity, and rosacea severity were the most significant predictors of DI. Having PPR increased the likelihood of detecting DI by 4.410 times, while obesity and rosacea severity increased this probability by 4.335 and 2.539 times, respectively.

There are two main limitations of this study. First, there is controversy about the SSSB method we used to determine *Demodex* density, which might be inadequate and give false negative results. Secondly, the sample size was relatively small. These limitations require more research, ideally involving larger patient groups and different methods for calculating *Demodex* density.

In conclusion, we identified obesity as a significant risk factor for *Demodex* infestation in rosacea patients. Therefore, evaluating obese rosacea patients for *Demodex* infestation may aid in the prompt formulation of effective treatment plans.

## AUTHOR CONTRIBUTIONS

Esra Kiratli Nalbant (EKN) formulated the study question, developed the study design, collected and analyzed data, and drafted manuscript. The other authors contributed the interpretation of the results and revised the manuscript. EKN had primary responsibility for final content. All authors read and approved the final manuscript.

## FUNDING INFORMATION

None.

## CONFLICT OF INTEREST STATEMENT

None.

## ETHICS STATEMENT

This study was approved by the Ethic Committee of Health Sciences University, Ankara Training and Research Hospital, Turkey. (protocol no:48865165‐020, date of approval:01/22/2018).

## Data Availability

The data that support the findings of this study are available from the corresponding author, upon reasonable request.
